# Editorial: Neuroimaging in psychiatry 2023: neurodegenerative disorders

**DOI:** 10.3389/fpsyt.2025.1653069

**Published:** 2025-07-09

**Authors:** Jannik Prasuhn, Prabesh Kanel

**Affiliations:** ^1^ Department of Neurology, University Medical Center Schleswig-Holstein, Lübeck, Germany; ^2^ Institute of Neurogenetics, University of Lübeck, Lübeck, Germany; ^3^ Center for Brain, Behavior and Metabolism, University of Lübeck, Lübeck, Germany; ^4^ Department of Neurology, Johns Hopkins University School of Medicine, Baltimore, MD, United States; ^5^ F.M. Kirby Research Center for Functional Brain Imaging, Kennedy Krieger Institute, Baltimore, MD, United States; ^6^ Department of Radiology, University of Michigan, Ann Arbor, MI, United States; ^7^ Morris K. Udall Center of Excellence for Parkinson’s Disease Research, University of Michigan, Ann Arbor, MI, United States; ^8^ Parkinson’s Foundation Research Center of Excellence, University of Michigan, Ann Arbor, MI, United States

**Keywords:** neuroimaging, psychiatry, neurology, dementia, neurodegeneration

Neurodegenerative disorders (NDs) represent a growing global health challenge. Despite decades of intense research, their etiologies remain elusive, and therapeutic options are largely symptomatic. However, neuroimaging continues to offer transformative insights, enhancing our understanding of these disorders from early pathophysiological mechanisms to the prediction of treatment responses. The Research Topic *“Neuroimaging in Psychiatry 2023: Neurodegenerative Disorders”* consolidates cutting-edge findings at the intersection of psychiatry, neurology, and neuroimaging science. The seven contributions featured in this Research Topic highlight novel methods, conceptual advances, and potential clinical applications that collectively advance the field.

A central theme emerging from the collected articles is the effort to translate imaging-derived biomarkers into clinically actionable insights. Prasuhn et al. offer a foundational review of advanced MRI techniques for assessing glymphatic system function. By integrating motion-sensitive diffusion imaging, dynamic contrast-enhanced MRI, and glucose-enhanced imaging, they provide a methodological blueprint for probing this clearance pathway *in vivo*. Glymphatic dysfunction is increasingly recognized as a shared mechanism underlying protein accumulation in Alzheimer’s (AD), Parkinson’s (PD), and Huntington’s disease (HD), and its imaging may serve as a convergent biomarker for therapeutic monitoring. This systems-level view positions glymphatic assessment as a promising biomarker not just of disease burden but potentially of treatment response, especially in trials targeting proteinopathies.

In parallel, Hata et al. address diagnostic accessibility through the lens of neurophysiology. Their original study demonstrates that portable EEG recordings, when coupled with vision transformer-based deep learning, can distinguish patients with AD from controls with high accuracy. The strength of their approach lies in its scalability and cost-effectiveness, offering a practical route toward early detection particularly in settings with limited access to advanced neuroimaging. Futhermore, the technology shows significant potential as a non-invasive screening method for identifying patient eligibility for disease-modifying therapies that require evidence of AD pathology.

This emphasis on accessible biomarkers aligns with a review by Tursini et al., which concluded that visual system electrophysiology can reveal a valuable biomarkers of central nervous system dysfunction. Deficits in the N95 wave of the electroretinogram (ERG) and the P100 wave of the visual evoked potential (VEP) are consistently reported across Alzheimer’s, Parkinson’s, and major depressive disorders reveals components implicated in retinal and cortical processing, respectively. These findings support a systems-level view in which peripheral and central visual pathways reflect broader neurodegenerative pathology.

Network-level insights are further elaborated by Cotta Ramusino et al., who investigate the relationship between fronto-limbic atrophy and neuropsychiatric symptoms in dementia. Their morphometric analyses link cortical thinning and subcortical volume reductions to behavioral manifestations such as apathy, agitation, and delusions. Notably, their stratified approach across AD, non-AD, and mild cognitive impairment (MCI) groups aligns with the broader aim of precision phenotyping in dementia care. These findings also resonate with broader efforts to move beyond categorical diagnosis, emphasizing a symptom-domain-based approach to NDs.

In a related vein, recent evidence from Mizuno et al. demonstrates that lower activation in the dorsomedial thalamus during a digit-symbol substitution task is associated with subjective cognitive decline (SCD), implicating early thalamic dysfunction as a potential contributor to cognitive vulnerability even before clinical impairment becomes apparent. While slower reaction times were associated with SCD severity, they were not directly related to brain activation or amlyoid-beta (Aβ) load. This work proposes that SCD symptoms acts as a mediating factor, suggesting that the influence of Aβ on declining neural and behavior function is channeled through the individual’s perception of cognitive decline. The work aligns with the broader thrust of network-sensitive neuroimaging, as disruptions in subcortical relay regions may precede and predict downstream cortical degradation.

Building on structural MRI, Ali et al. harness convolutional neural networks (CNNs) to classify and stage dementia. The authors developed a two-stage CNN that first identifies dementia and then subtypes it into mild, moderate, and severe disease stages using transfer learning. This approach with a multistage CNN framework optimized classification performance across multiple datasets. This study shows accuracies exceeding 95%, suggesting that machine-learning models can bridge the gap between expert interpretation and automated, scalable diagnostics. These deep learning-based models represent a critical advance in integrating imaging data with clinical decision tools. Crucially, their methodologies mirror real-world diagnostic cascades—first detecting abnormality, then refining into subcategories. The potential to embed such models into clinical workflows, radiology pipelines, or even cloud-based teleradiology systems represents a step toward equitable dementia care.

Doganyigit et al. provide important longitudinal context by identifying predictors of cognitive decline in Alzheimer’s disease. Their findings indicate that the combination of encoding deficits and temporal lobe atrophy is more robust predictor of cognitive decline, rather than retrieval failure, as measured by the Mini-Mental State Examination (MMSE). These results reinforce the clinical utility of combining cognitive and imaging biomarkers, particularly when aiming to predict disease progression rather than static diagnosis. Interestingly, their findings validate a key hypothesis that early encoding impairments may represent a more sensitive marker of disease trajectory—potentially influencing clinical trial design, patient monitoring, and stratified intervention approaches.

Taken together, the contributions in this Research Topic form an interconnected narrative that spans microscopic physiology, circuit-level dysfunction, behavioral syndromes, and computational diagnostics. The convergence of methods—ranging from portable EEG and visual electrophysiology to high-resolution MRI and use of machine learning techniques like CNN-based analytics—underscores the field’s dynamic methodological evolution. More importantly, the integration of these modalities within shared disease frameworks highlights a growing consensus: that neuroimaging, when thoughtfully deployed, can serve as a linchpin for precision diagnostics, stratified therapeutics, and early intervention in neurodegenerative disease.

We hope this Research Topic inspires further cross-disciplinary research and accelerates the translation of neuroimaging science into meaningful clinical outcomes. It is only through such integrated, multiscale efforts that the field will realize the full potential of imaging as both a diagnostic and therapeutic compass in the evolving landscape of personalized care for patients with NDs.

